# High burden of RSV and influenza in patients presenting with suspected pneumonia in the emergency room of a German tertiary hospital in fall of 2022

**DOI:** 10.1007/s15010-023-02069-w

**Published:** 2023-07-04

**Authors:** Marlene Ettemeyer, Maria Florey, Konstantin Tanida, Johannes Jochum, Ulf Schulze-Sturm, Marc Lütgehetmann, Michael Baehr, Marylyn M. Addo, Stefan Schmiedel, Holger Rohde, Till Koch

**Affiliations:** 1 Division of Infectious Diseases, First Department of Medicine, University Medical Center Hamburg-Eppendorf, Hamburg, Germany; 2 German Centre for Infection Research (DZIF), Partner Site Hamburg-Lübeck-Borstel-Riems, Riems, Germany; 3 Antibiotic Stewardship Team, Pharmacy of the University Medical Center Hamburg-Eppendorf, Hamburg, Germany; 4 Department of Pediatrics, University Medical Center Hamburg-Eppendorf, Hamburg, Germany; 5 Institute of Medical Microbiology, Virology and Hygiene, University Medical Center Hamburg‐Eppendorf, Hamburg, Germany; 6 Institute for Infection Research and Vaccine Development, University Medical Center Hamburg-Eppendorf, Hamburg, Germany; 7 Department of Tropical Medicine, Bernhard Nocht Institute for Tropical Medicine and First Department of Medicine, University Medical Center Hamburg-Eppendorf, Hamburg, Germany

**Keywords:** Pneumonia, Diagnostics, RSV, Influenza, Emergency

## Abstract

**Purpose:**

Bacterial pneumonia, a major cause of respiratory tract infections (RTI), can be challenging to diagnose and to treat adequately, especially when seasonal viral pathogens co-circulate. The aim of this study was to give a real-world snapshot of the burden of respiratory disease and treatment choices in the emergency department (ED) of a tertiary care hospital in Germany in the fall of 2022.

**Methods:**

Anonymized analysis of a quality control initiative that prospectively documented all patients presenting to our ED with symptoms suggestive of RTI from Nov 7th to Dec 18th, 2022.

**Results:**

243 patients were followed at the time of their ED attendance. Clinical, laboratory and radiographic examination was performed in 92% of patients (224/243). Microbiological work-up to identify causative pathogens including blood cultures, sputum or urine-antigen tests were performed in 55% of patients (*n* = 134). Detection of viral pathogens increased during the study period from 7 to 31 cases per week, while bacterial pneumonias, respiratory tract infections without detection of a viral pathogen and non-infectious etiologies remained stable. A high burden of bacterial and viral co-infections became apparent (16%, 38/243), and co-administration of antibiotic and antiviral treatments was observed (14%, *n* = 35/243). 17% of patients (41/243) received antibiotic coverage without a diagnosis of a bacterial etiology.

**Conclusion:**

During the fall of 2022, the burden of RTI caused by detectable viral pathogens increased unusually early. Rapid and unexpected changes in pathogen distribution highlight the need for targeted diagnostics to improve the quality of RTI management in the ED.

Every fall and winter, symptoms suggestive of respiratory tract infections (RTI) are among the leading causes for patients to seek medical care in emergency departments. One of the most important differential diagnoses is pneumonia, which requires swift diagnosis and treatment initiation. Bacterial pneumonia in particular has a variable disease severity that can range from mild infections treated in the outpatient setting to severe respiratory failure demanding treatment in the intensive care unit. Bacterial pneumonia can, however, be challenging to diagnose, since the diagnosis does not rely solely on one finding but instead requires a complete “picture” of clinical, radiographic, laboratory and microbiological findings that should be employed according to national [1] and international [2] guidelines. Especially if etiological evaluation is incomplete, difficulties arise in differentiating bacterial from other etiologies of respiratory disease (e.g. seasonal viruses or noninfectious etiologies that can cause similar symptoms). As a consequence, inappropriate diagnostic workup increases the risk of “over-treatment”, i.e., administration of unnecessary antimicrobial treatments. The excess use of antibiotics might expose patients to avoidable adverse events and supports the emergence of resistant bacteria [3].

For the clinician on the ground, the index of suspicion for specific respiratory pathogens depends largely on the seasonality of certain infectious agents such as influenza virus. In past decades, the detection of these pathogens has followed a fairly predictable pattern with a typical influenza season in Germany ranging from November to March and a peak of influenza detection rates in February [4]. However, this seasonality has changed after the widespread adaption of respiratory precautions such as masking during the recent COVID-19 pandemic. While there was almost no influenza and RSV virus detected in Germany in the season 2020/2021 [5, 6], the following season of 2021/2022 was characterized by a relatively high burden of disease [7]. In late 2022, we observed an unusually early increase in the detection of viral respiratory pathogens. Availability and access to molecular testing capacities set up during the recent COVID-19 pandemic have paved the way for the implementation of routine testing strategies for broader detection of common viral respiratory pathogens in airway specimens. For example in our institution, respiratory specimens sent for SARS-CoV-2 testing were systematically tested for influenza A and B, as well as a respiratory syncytial virus (RSV).

At the dawn of the current wave of viral airway infections, we were recruiting a cohort of patients for a study on community-acquired pneumonia (CAP) in 2022 (Ethikkommission Hamburg, Bearb.Nr. 2022-100835-BO-ff). When the first cases of viral infections started to be detected unusually early compared to previous years, we initiated an assessment of current diagnoses and outcomes of patients presenting to the emergency department (ED) with suspicion of pneumonia. The aim was to quantify the burden of disease of bacterial, viral and non-infectious causes in this patient population, as well as diagnostic testing performed and treatment initiated in a real-world setting.

Since all data was collected as a quality control initiative by our local antibiotic stewardship (ABS) team and retrieved anonymously, no specific enrolment of study participants was necessary, in accordance with data protection standards. Data of patients who presented to the ED of our tertiary hospital were prospectively collected over 6 weeks from Nov 7th to Dec 18th, 2022 (weeks 45 to 50) by a daily screening of the ED dashboard, chart review and correspondence with the ED staff. All adult patients (≥ 18 years of age) who presented with suspected pneumonia were documented. The assessment and stratification, e.g. as suspected or confirmed pneumonia, was made by the attending physician. Signs and symptoms that prompted suspicion of pneumonia included respiratory symptoms, such as cough or dyspnea, as well as general symptoms of fever, malaise, headaches or myalgia and typical signs in the physical examination, such as pathological auscultation. Laboratory measures such as Leukocyte count or C-reactive protein (CRP), were assessed as well as radiological findings in chest radiographies or computed tomography (CT) scans. Lastly, microbiological findings in sputum specimens, urine (legionella antigen, pneumococcal antigen) and blood cultures were included. All data were anonymized before retrieval.

Patients were followed for the time of their attendance in the ED through observation of the electronic medical record by the ABS team. The method of prospective documentation was chosen in opposition to a retrospective observation of electronic health records, since retrospective analysis of the documented International Classification of Diseases (ICD)-codes alone had previously led to a skewed picture of the burden of disease (unpublished data). Prospective data collection provided the opportunity to receive an accurate impression of the diagnostic tests that were performed, the epidemiology of causative pathogens and the treatments that were administered. For this purpose, the following parameters were recorded: type of diagnostic tests performed, diagnosis at the end of ED attendance and antimicrobial therapy initiated in the ED. Among the diagnostic tests, a complete clinical examination was recorded if documentation of physical examination including respiratory examination, respiratory rate and oxygen saturation was available in the patient’s electronic health record. Laboratory examination was recorded if the parameters of absolute Leucocyte count, creatinine and CRP were analyzed. Radiographic findings were recorded if the patient received either a chest X-ray or a computed tomography of the chest. In cases where a radiographic examination was performed, we categorized the findings into infiltrates typical for pneumonia (“pneumonic” infiltrates), suspected infiltrates or no infiltrates. Microbiological workup was recorded if analysis of sputum samples, throat swabs, urine for pneumococcal or legionella antigen, or blood cultures were analyzed according to in-house standards.

We stratified the patients according to suspected etiology and antimicrobial agent as described in Table 1. Patients were considered to have an upper (URTI) or lower respiratory tract infection (LRTI) as their primary diagnosis as determined by the treating clinicians. Bacterial pneumonia was diagnosed if the clinical, radiological, laboratory and microbiological findings were consistent with the diagnosis of bacterial pneumonia. If patients were transferred from another hospital or readmitted after a recent discharge (≤ 3 months), pneumonia was defined as healthcare associated (HAP). Patients were considered to have a viral respiratory tract infection if a respiratory specimen (sputum or throat swab) tested positive for one of the following pathogens via PCR: Influenza A or B, SARS-CoV-2 or RSV. Patients were classified as “noninfectious cause suspected” if the abovementioned criteria did not fit a bacterial or a viral etiology and if there was an alternative explanation for the symptoms, with the two most common being cardiac decompensation and exacerbated chronic obstructive pulmonary disease (COPD). The latter could well be due to viruses that were not routinely tested for (e.g. rhinovirus, adenovirus, parainfluenza, etc.) due to the lack of consequences in management resulting from a positive test. It is, therefore, possible that some cases classified as “Noninfectious cause suspected, no infectious pathogen detected” were actually caused by viruses that were undetected. In some cases, patients had more than one diagnosis, e.g. cardiac decompensation and a virus detected in a respiratory specimen. In this case, the primary etiology was determined by the treating physician with the other etiology being recorded as secondary. As this was an observational study that aimed to capture a “real-world” impression of diagnostic and treatment choices, not all patients underwent the same evaluation. Instead, the tests performed to determine the responsible etiology were chosen at the treating physician’s discretion. We recorded the treatment administered as an antibiotic or as antiviral if treatment was initiated in the ED and patients received at least a 24-h course of treatment.

**Table 1:** Stratification of study population according to the etiology suspected by the treating clinician, pathogens detected and antimicrobial agents administered

Stratification of study population		
Etiology, as suspected by clinician	Pathogens detected	Antimicrobial agents administered
LRTI: Community acquired (CAP) Healthcare associated (HAP)	no viral pathogen detected	Antiviral
		Antibiotic
URTI	no viral pathogen detected	Antiviral
		Antibiotic
LRTI or URTI	viral pathogen detected: Influenza SARS-CoV-2 RSV	Antiviral
		Antibiotic
Noninfectious cause suspected: Exacerbated COPD Cardiac decompensation Other	no infectious pathogen detected	Antiviral
		Antibiotic

*LRTI* lower respiratory tract infection, *URTI* upper respiratory tract infection

In the observation period, a total of 243 patients had pneumonia included in their differential diagnoses, of which 162 (67%) were admitted as inpatients. Figure 1 gives an overview of diagnostic tests performed, primary etiologies recorded and treatments administered. Empiric antibiotic coverage of pneumonia was chosen according to local guidelines and depended on the severity of the disease, the setting of acquisition of pneumonia (CAP vs. HAP) and underlying conditions. Antibiotic agents included oral amoxicillin for outpatient treatment of pneumonia without risk factors for progression to severe disease, intravenous ampicillin/sulbactam for inpatients with CAP or HAP and piperacillin/tazobactam for patients with increased risk for multi-drug resistant organisms due to underlying conditions, e.g. COPD. Antiviral treatment included nirmatrelvir/ritonavir and remdesivir for the treatment of SARS-CoV-2 infection as well as oseltamivir for the treatment of Influenza infections.

**Fig. 1:**
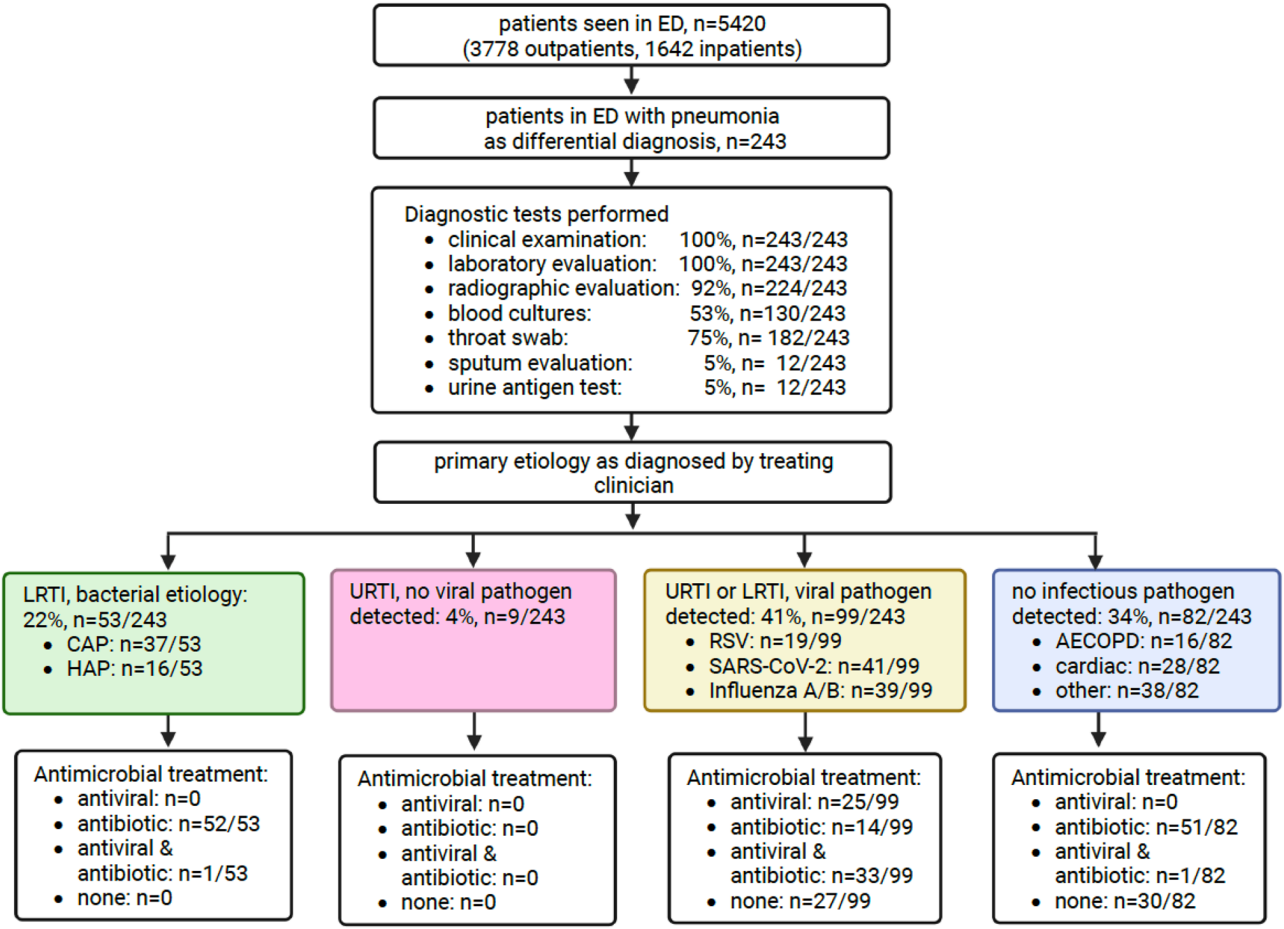
Study flow chart and results. From all patients analyzed in the study period, diagnostic tests performed are reported in the third row. Primary etiologies as determined by the treating physician are reported in the fifth row. Treatments administered to the respective etiologies are reported in the sixth row. LRTI: lower respiratory tract infections, URTI: upper respiratory tract infections

A more detailed demonstration of diagnostic tests performed of clinical, radiographic, laboratory and microbiological tests in the total analyzed cohort is shown in Fig. 2A, while diagnostic tests performed in those patients with bacterial pneumonia as primary etiology are shown in Fig. 2B. Clinical and laboratory examination (including total Leucocyte count and CRP) were performed in 100% of patients and radiographic tests (either chest radiography or computed tomography of the chest) in 92% (*n* = 224/243) and 98% (*n* = 52/53) of the total analyzed population, and of the group with pneumonia as a primary diagnosis at the end of ED stay, respectively. Microbiological assessment was performed in 84% (*n* = 203/243) of patients. Specifically, blood cultures were drawn in 53% of patients in the total analyzed population, while urine and sputum were analyzed in 5%, respectively in the total analyzed population. These numbers were slightly higher in the group that had bacterial pneumonia as the primary diagnosis (blood cultures 62%, urine 13%, sputum 11%). In 75% of patients (*n* = 182), a throat swab was analyzed for viral pathogens.

**Fig. 2:**
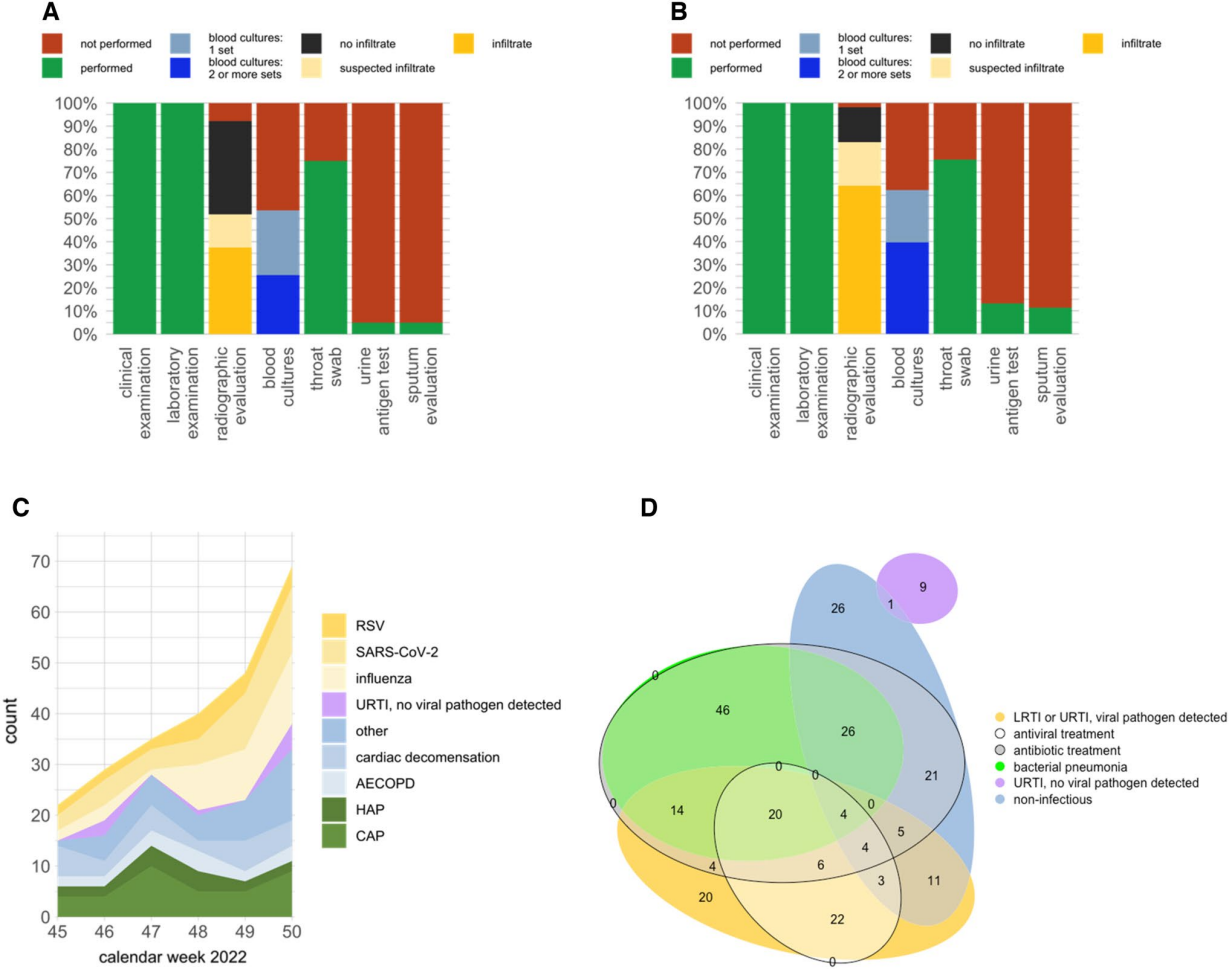
Diagnostic tests, etiologies and antimicrobial treatment administered in patients with pneumonia as differential diagnosis. **A, B** Column diagrams demonstrating diagnostic utilization of clinical, laboratory, radiographic and microbiological tests in patients with suspected pneumonia, i.e., the total analyzed population (**A**) and in those with bacterial pneumonia as the primary diagnosis at the end of ED visit (**B**). **C** Stacked Area graph of determined primary etiologies over time. Bacterial LRTI are shown in different shades of green, URTI in which no pathogen was detected in pink, URTI or LRTI in which a viral pathogen was detected in different shades of yellow and non-infectious etiologies as shades of blue. **D** Euler diagram showing overlapping proportionally weighed ellipses of determined etiologies and administered treatments throughout the study period. The etiologies are depicted as colored zones without borders (green: LRTI, bacterial pneumonia; yellow: LRTI or URTI, viral pathogen detected; violet: URTI, no viral pathogen detected; blue: non-infectious); the antimicrobial treatments administered are depicted as grey zones with edged borders (antiviral: white, antibiotic: grey). The numbers depicted represent the number of cases where the respective attributes were recorded. *LRTI* lower respiratory tract infections, *URTI* upper respiratory tract infections

An overview of the development over time of the primary etiologies is given in Fig. 2C via a stacked area graph. During our study period, an increased detection of viral pathogens as potential causative agents of respiratory infections could be observed. While there were 7 (33%, *n* = 7/22) cases of viral infections recorded as primary etiology in week 45, the number increased to 31 (48%, *n* = 31/65) by week 50. Bacterial pathogens could be detected in only one case of bacterial LRTI. However, a complete microbiological assessment (≥ 2 pairs of blood cultures, throat swab, sputum and urine) was performed in only five out of 53 patients with a diagnosed bacterial pneumonia, so the majority of these diagnoses were determined clinically. While the number of bacterial pneumonias remained stable during the study period, cases where no infectious pathogen was detected increased in the last week of observation.

Figure 2D shows an Euler diagram that demonstrates the overlapping of etiologies and administered treatments throughout the study period. There was substantial overlapping of etiologies and treatments. All patients that were diagnosed with bacterial pneumonia as the primary diagnosis (*n* = 53) were treated with an antibiotic, while 58 out of 99 patients (59%) with a viral infection as the primary diagnosis were treated with an antiviral agent. Out of 80 patients who were diagnosed with SARS-CoV-2 or influenza virus as the primary diagnosis, 23 (29%) did not receive an antiviral medication. In the absence of a bacterial etiology, 41 out of the total 243 patients (17%) received antibiotic coverage, of these, in 10 patients a viral pathogen could be detected, 22 were diagnosed with a non-infectious cause and 9 with both. In relation to the 151 patients that received antibiotic treatment, these 41 patients indicate an “unnecessary” use of antibiotics in 27% of patients.

A minority of patients with bacterial pneumonia as the suspected primary etiology did not show infiltrates in their radiographic imaging. This could be due to the fact that infiltrates had not yet fully developed in an early cause of disease or that the sensitivity of plain chest X-ray is comparatively low [8], compared to other modalities like computed tomography.

Not all patients with viral pathogens detected were treated with an antiviral agent, which might be explained in part by the fact that there are only limited well-established treatment options for RSV available and therapies of uncertain use, such as Ribavirin, would not routinely be administered in immunocompetent hosts. Detection of SARS-CoV-2 or influenza virus would likewise not automatically lead to the administration of antiviral medication, since early treatment (i.e., in the first few days after symptom onset) of these viruses is recommended by current guidelines [9, 10].

The overuse of antibiotics is a common phenomenon in the ED and one that cannot be entirely avoided since a potential overuse of antibiotics must be weighed against the potential harm from delaying treatment of serious bacterial infections in the frenzy of day-to-day ED reality. In this regard, overuse of antibiotics in 27% of the cases seems to be an acceptable amount.

This study has several limitations. First, the diagnostic methods employed were not the same for every patient but were chosen according to the respective physician’s discretion. Second, the determination of the final diagnoses relied on the judgement of the treating physicians in the ED. An independent evaluation of the primary diagnosis, e.g. at the end of the hospital stay, was not performed. Third, this study represents only a small timeframe, albeit one in which the viral causes of disease increased substantially over time. The study’s strengths are its observational nature that depicts a *real-world* impression of diagnostic and treatment choices.

Clinical, laboratory and radiographic assessment were frequently utilized in the assessment of patients. However, the rate of microbiological sampling documented in our study seems low. While throat swabs and blood cultures were obtained in the majority of patients, urine and sputum were only collected in a small fraction of cases, even when bacterial pneumonia was the primary diagnosis. However, even in cases where microbiological sampling was performed as recommended by guidelines [1, 2], a responsible bacterial pathogen could be detected in only one case, in which the empiric treatment administered covered the causative organism sufficiently. In patients that have a low risk of progression to severe RTI, one could argue that only minimal microbiological assessment would be necessary [11]. On the other hand, a sufficient microbiological sampling could offer the possibility to detect causative pathogens more frequently and allow for targeted antibiotic therapy [12].

Further studies and subsequent antimicrobial stewardship interventions could help to identify challenges in obtaining microbiological samples in the emergency department and clarify which amount of microbiological sampling would be best for which patient population. During the intervention period, viral etiologies of respiratory symptoms increased markedly while bacterial etiologies remained stable. There was a substantial overlap of etiologies and many non-bacterial etiologies also received antibiotic coverage.

In summary, this report delivers a *real-world* snapshot of the burden of respiratory disease and *de facto* treatment choices, highlighting diagnostic and therapeutic challenges in a tertiary hospital in Germany in the fall of 2022.

## Data Availability

Raw data were generated at University Medical Center Hamburg-Eppendorf within the internal electronic health system. Derived data supporting the findings of this study are available from the corresponding author T. Koch on request.
